# Simulator-Assisted Training of Abomasal Surgery—A Pilot Study Using Blended Learning and Face-to-Face Teaching

**DOI:** 10.3390/ani13243822

**Published:** 2023-12-11

**Authors:** Julia Muehlberg, Andrea Tipold, Maike Heppelmann, Sandra Wissing

**Affiliations:** 1 Clinical Skills Lab, Centre for E-Learning, Didactics and Training Research, University of Veterinary Medicine Hannover, 30173 Hannover, Germany; sandra.wissing@tiho-hannover.de; 2 Clinic for Small Animals, Neurology, University of Veterinary Medicine Hannover, 30559 Hannover, Germany; andrea.tipold@tiho-hannover.de; 3 Clinic for Cattle, University of Veterinary Medicine Hannover, 30173 Hannover, Germany; maike.heppelmann@tiho-hannover.de

**Keywords:** veterinary education, clinical skills, blended learning, simulation, self-efficacy, abomasal displacement

## Abstract

**Simple Summary:**

Learning surgical skills on simulators is being integrated into the curriculum of veterinary students. While education has traditionally been face-to-face only, the combination of face-to-face and online teaching is described as blended learning. It is tested for its suitability for learning a bovine surgical technique. Two simulators were developed for the study. While one group of students trained on the simulator in a traditional face-to-face setting, the other group of students completed online video training. The skills learned were applied in-person. The simulators used were tested and evaluated by students and practicing veterinarians and were well accepted and rated as suitable. The study indicates that blended learning can be a suitable alternative to traditional face-to-face teaching. In addition, the students were, according to their own assessment, more confident in performing the practiced skills after the exercise.

**Abstract:**

Two stimulators were developed, one simplified and one realistic, in the present study for learning abomasal surgery for veterinary students. The simulators were tested in a pilot study: The upcoming blended learning format was compared with traditional face-to-face teaching. A total of 21 5th-year students participated in the study. While one group learned the surgical technique in traditional face-to-face simulator training, the second group completed interactive video training asynchronously. Afterwards, skills were examined in person. The results showed that the different groups did not lead to different performance results. Participation in the study increased self-assessment of skills by an average of about 7 of 36 points, as well as the learning success and motivation of students in both groups. The simulators developed were well liked by the students and rated as appropriate by 12 practicing bovine veterinarians. The pilot study indicates that blended learning could be a suitable alternative to traditional face-to-face teaching. This should be followed by further research to support the use of blended learning in the veterinary education of clinical skills.

## 1. Introduction

Abomasal displacement (AD) is one of the most common diseases in cattle. As the treatment for this is usually surgical, performing abomasal surgery should be a basic competency of prospective bovine practitioners [1]. Since it has not yet been possible for all students to learn the surgical technique practically at the University of Veterinary Medicine Hannover, Foundation (TiHo), Hannover, Germany, the aim of the present study was to test the possibility of learning the technique on simulators and applying it independently by developing two simulators for this surgical technique. The scenario was limited to the far more common left-sided AD and the Dirksen procedure. The laparotomy on the right flank with omentopexy (Dirksen method) makes the operation (Op) method versatile—it is suitable for both left- and right-sided AD. In addition, a complete exploration of the peritoneal cavity allows for a diagnosis to be made [2,3,4].

The Clinical Skills Lab (CSL) at the TiHo was established to introduce students to the basic and advanced practical skills of veterinary medicine during their studies. These skills can be learned, repeated, and deepened in a protective setting. Afterwards, students should be able to transfer and apply these skills to live animals. During the COVID-19 pandemic, face-to-face teaching at the TiHo was temporarily interrupted. Subsequently, the CSL provided students with material for learning clinical scenarios on the learning platform Moodle. With the lifting of the pandemic restrictions, the concept of blended learning has become established at the CSL. Here, online teaching and face-to-face teaching are mixed: after asynchronous online preparation at home, the time spent in face-to-face learning can be effectively used to learn and consolidate clinical practical skills [5,6,7,8]. The model designed by Lewis and Orton [9] demonstrates that knowledge can be acquired at one’s own speed during the online preparation phase. In the subsequent classroom instruction, the focus is on applying those already acquired skills. Afterwards, the knowledge is revised and deepened online [9]. The teaching method of blended learning is considered a further development of online teaching. Since the introduction of pure online teaching, the spatial separation and the resulting lack of social interaction have proved to be the main disadvantages. This social component is supplemented in blended learning by the in-person practices [10,11].

To test the developed simulators, the present study compared blended learning with regular face-to-face teaching. Current studies demonstrated an increase in initial training success in the blended learning format [12,13,14,15]. The transferability of these findings should be tested for complex skills such as performing abomasal surgery.

In addition to mastering clinical skills, a realistic self-assessment of skills is important in the daily work of practicing veterinarians. Therefore, self-assessment of skills in relation to abomasal surgery was further investigated. The concept of self-efficacy describes the assessment of one’s own skills and the resulting influence on awareness and action [16,17]. People with high self-efficacy are confident in their skills and approach challenges with a positive attitude. This increases success and reduces stress at the same time. In contrast, people with low self-efficacy lack a sense of security and intrinsic motivation to solve problems [16,17]. Studies have been able to demonstrate the increase in self-efficacy in terms of skills [18,19,20,21,22,23,24,25]. Therefore, our study focused on testing the hypothesis of an increase in students’ self-assessment of skills. In this paper, this is compared with their examination performance.

In summary, the present study examined the following questions: How are the simulators evaluated overall and for their use in teaching? Is the method of blended learning an alternative to traditional face-to-face teaching for complex clinical skills? Can the self-assessment of the skills of the students be significantly increased, and how accurate is the assessment?

## 2. Materials and Methods

### 2.1. Data Privacy

The study was conducted based on the data privacy statement (Datenschutzbestimmung) Art. 6 I 1 lit. e i.V.m. 89 DSGVO, § 3 I 1 Nr. 1 NHG, § 13 NDSG and was reviewed and approved by the Data Protection Officer of the TiHo before the start of the study. An ethical evaluation and approval were granted by the Commission for Research Ethics of the TiHo under the number 23-PK-03.

### 2.2. Developed Simulators

In the present study, a simplified and realistic life-size simulator was developed for learning the Dirksen surgical technique for left-sided abomasal displacement in the CSL of the TiHo.

#### 2.2.1. Simplified Simulator (Simulator 1)

**Figure 1:**
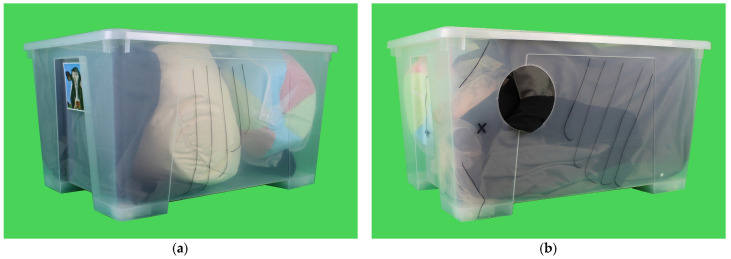
Simplified simulator: (**a**) left view and (**b**) right view. Transparent plastic box with replicas of greater omentum, omasum, intestine, rumen, and abomasum for learning the surgical technique for left-sided abomasal displacement.

The simplified simulator (Figure 1) provides a three-dimensional view and exploration of the basic anatomical structures of the bovine peritoneal cavity. The surgical steps of “Identifying the abomasum”, “Degassing the abomasum”, and “Repositioning the abomasum” can be performed with Simulator 1. Two different suture pads can be used outside the simulator for the steps “Suture plate to greater omentum” and “Suture button to abdominal wall”.

The simplified simulator is made from easily accessible materials. The basic structure is a plastic box with a lid measuring 78 × 56 × 43 cm. The box is transparent from all sides and represents the peritoneal cavity of a cow. Head, ribs, and tail are drawn on the box for topographical orientation. On the right side of the box, a circular cutout with a diameter of 20 cm allows access for the operation.

The box contains replicas of the greater omentum, omasum, intestine, rumen, and abomasum. The organs are made from the following materials: The greater omentum with its caudal edge is simulated by a curtain attached to the lid with Velcro tape. The omasum is represented by a soft football. The intestinal convolution is sewn from different pairs of tights onto tulle fabric, simulating the mesentery. The appendix can be varied in its filling state by means of an inflated pool noodle. The simulated rumen is represented by a bean bag. The centerpiece of the simulator is the simulated abomasum. An inflatable beach ball is integrated into the greater omentum, which is held in place by a mesh laundry bag and can be moved in its position.

#### 2.2.2. Realistic Simulator (Simulator 2)

**Figure 2:**
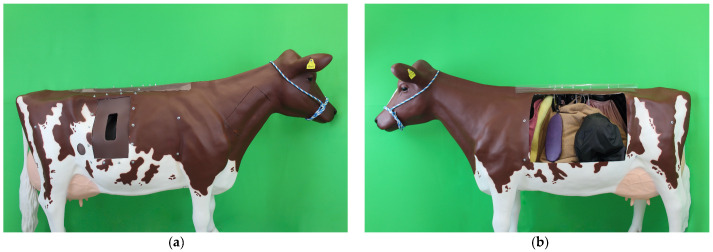
Realistic simulator: (**a**) right view of surgical access and (**b**) left view of abdominal organs. Life-size three-dimensional cow model with replicas of most abdominal organs. For exploring and learning the surgical technique for left-sided abomasal displacement.

The realistic simulator (Figure 2) is life-size. The basic setup is a three-dimensional cow model (Deko mit Pfiff International, Berlin, Germany).

**Figure 3:**
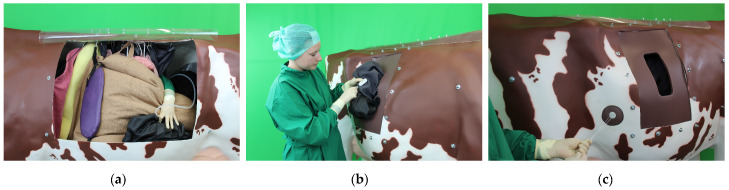
Surgical steps performed on the realistic simulator: (**a**) abomasal degasification, (**b**) suturing of the plate, and (**c**) suturing of the button.

All steps of the Dirksen surgical method for left-sided AD can be performed by using the realistic simulator (Figure 3). In addition, the integration of the simulated organs in the physiological position allows for a realistic exploration of the peritoneal cavity.

The cow model has different fenestrations: The surgical access is provided by an approximately 25 × 50 cm fenestration on the right side caudal to the costal arch below the lumbar transverse processes. A silicone insert is incorporated into this fenestration. A suture pad is integrated into another circular cutout on the right side, with a diameter of about 9 cm. This cutout is located two hand lengths above the knee crease, in a straight line under the cranial edge of the model’s point of hip. The suture pad consists of two different layers: a foam layer that faces the inside of the model and a brown-colored silicone layer that is visible from the outside. A window of approximately 60 × 70 cm on the left side of the chest and abdomen is covered by a transparent PVC foil and allows for a permanent view into the peritoneal cavity.

The interior of the simulator is covered with epoxy resin and contains the following replicated organs: diaphragm, liver, gall bladder, greater omentum, omasum, rumen with reticulum, spleen, abomasum, intestine convolute, left kidney, and uterus.

The materials were chosen to feel as close to reality as possible. To make the appearance look more realistic, ear tags and a knitted halter were added to the cow model.

**Figure 4:**
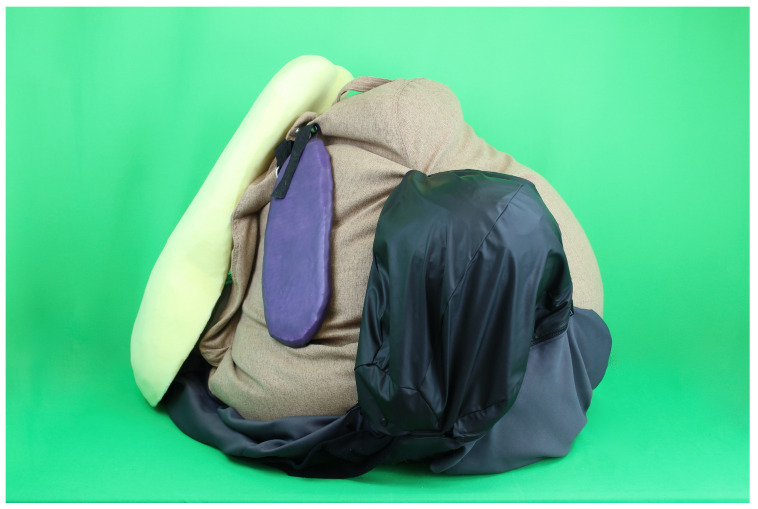
Left view of the organ replicas of the realistic simulator: organ replica of liver (yellow), spleen (purple), rumen (brown), and abomasum (black).

The diaphragm made from pink corduroy fabric serves as a barrier between the chest and peritoneal cavity. The replica liver (Figure 4) is attached to the diaphragm. The anatomical structure of the liver is molded from a foam block. The gallbladder is integrated into the foam in the form of a water-filled balloon.

The digestive apparatus is covered by a large net, which is simulated by means of net curtain fabric. A beanbag filled with granules and an only slightly inflated exercise ball represent the rumen (Figure 4). In this way, the layering of the rumen with a dorsal gas bubble and a pressable rumen structure is created. With the aid of a zipper, the filling state of the rumen can be varied. Moreover, the reticulum is sewn into the beanbag, which is filled with a magnet. The omasum, located between the rumen and the liver, is represented by a football. It is movably attached to the bean bag with the help of a washing net. The abomasum (Figure 4), which comes after the digestive system, is made of rainwear fabric with a smooth surface. Zippers are integrated into the oval abomasum. On the one hand, these zippers ensure that the organ is attached to the greater omentum, and on the other hand, they allow the abomasum to be filled. The position of the abomasum in the peritoneal cavity of the model is variable. The venting of the abomasum is made possible by a balloon covered with tape. To represent the pylorus, a small foam ball is incorporated into the mold. The intestinal convolution contains replicas of the duodenum, jejunum, ileum, appendix, and colon. These are made from different types of tights and sewn onto the mesentery simulated by fabric. The loops are filled to varying degrees with wadding and padding film. The left kidney is modeled and cast in silicone. The kidney mold of the Ultrasonic Cow Simulator by Dr. Silja Brombacher-Steiert is used to shape the kidney [19]. The spleen (Figure 4) is incorporated into the model close to the beanbag on the left side wall. The organ replica is molded from Styrofoam and covered with castable rubber and colored purple. Letter-clips in the pelvic cavity of the cow model allow for the use of different uterine simulators.

The abdominal organs are attached to the cow model with eyelets, ropes, and carabiners.

### 2.3. Study Design

#### 2.3.1. Study with Students in Their Clinical Year

**Figure 5:**
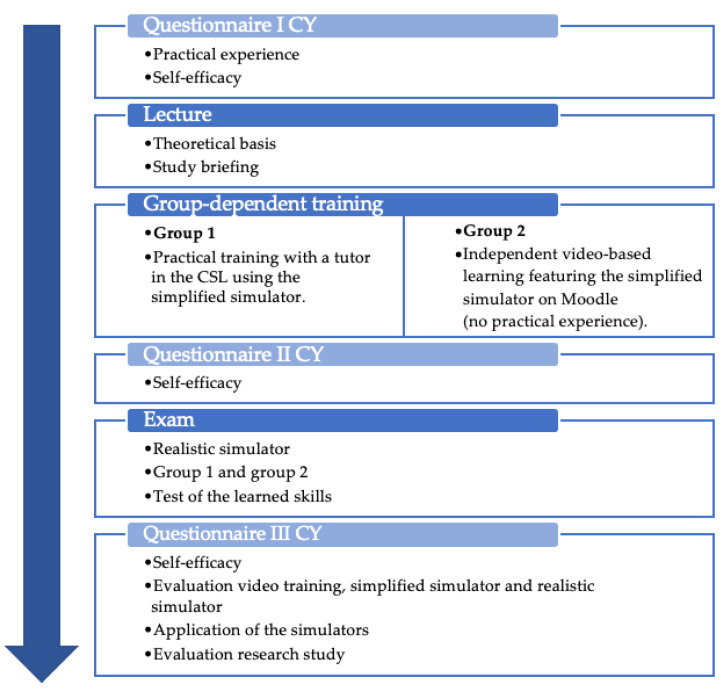
Procedure for students in their clinical year (CY).

In total, 21 veterinary students were involved in the study (Figure 5). The study was realized in three cycles in March, May, and July 2023 in the 5th year of veterinary studies within the clinical year (CY). The participants completed their clinical year cycle at the Clinic for Cattle of the TiHo.

At the beginning of the study, the questionnaire I CY (see Supplementary Document S1) was used to record the level of experience of the participants in relation to bovine abomasal surgery. Students were classified as “experienced” if they independently performed at least one-to-five abomasal surgeries, assisted in at least six-to-ten abomasal surgeries by laparotomy or by endoscopy, or assisted in at least one-to-five abomasal surgeries by laparotomy and at least one-to-five abomasal surgeries by endoscopy. In the first questionnaire, participants were also asked to rate their self-assessment of skills regarding abomasal surgery.

Subsequently, all participants received a 60-min lecture, which served as a theoretical basis. The presentation focused on the anatomy of the peritoneal cavity and its exploration. Furthermore, the surgical technique for a left-sided AD using the Dirksen method was presented.

After the lecture, the participants were divided into two groups: “experienced” and “inexperienced” students were equally split between Group 1 and Group 2. The randomized assignment into the groups was performed with the statistical software R, version 4.2.1 (R Core Team (2022). R: A Language and Environment for Statistical Computing. R Foundation for Statistical Computing, Vienna, Austria) and its combinat package (Chasalow S (2012)). combinat: combinatorics utilities. R package version, 0.0-8, <https://CRAN.R-project.org/package=combinat, accessed on 16 February 2023>.

Group 1 trained the surgical technique in face-to-face sessions at the CSL on the simplified simulator in a 60-min training course. Group 2 completed the training asynchronously via the platform Moodle. A video was created for this purpose. It showed a Dirksen abomasal resection in a left-sided AD on the simplified simulator. The video was enriched with H5P modules. This allowed for interactive responses to multiple-choice, single-choice, and matching questions. Students were also able to click within the video for more detailed information and visuals. The complete processing of the video and the questions was checked in Moodle. The video was 10:27 min in length, not including pauses for interaction, and students could watch it multiple times. The face-to-face and video training included instruction in the (incomplete) exploration of the peritoneal cavity and performance of the Dirksen abomasal surgery for left-sided AD. The surgical technique was split into the following steps:

- Operation preparation;
- Identifying the abomasum during exploration;
- Abomasal degasification;
- Abomasum repositioning;
- Correct suturing of the plate in the greater omentum;
- Correct fixation of the plate to the abdominal wall, using a button.

Immediately following the group-based training, participants again answered self-assessment-of-skills questions in Questionnaire II CY (see Supplementary Document S2).

This was followed by a test of the learned clinical skills in the form of an Objective Structured Clinical Examination (OSCE) on the realistic Simulator 2. The OSCE was realized one-to-four days after the training, depending on the group and the cycle. The task was to perform an abomasal operation on a left-sided AD in cattle without assistance. The key point was the surgical technique using the Dirksen method so that the opening and closure of the peritoneal cavity were not practically demonstrated by the students. The time limit for completing the scenario was 25 min. The evaluation was based on an objective checklist (see Supplementary Document S5). It contains the individual steps of the required operation, as well as their correct performance. The evaluators were independent veterinarians that had been trained both in the topic and for their role as evaluators. They were not involved in the practical training or group allocation. The evaluators could not draw any conclusions about group membership.

At the end of the study, participants completed Questionnaire III (CY) immediately after the OSCE. This included the self-assessment of skills and evaluation of the study. The video training, Simulator 1 and Simulator 2, and their application were rated.

#### 2.3.2. Evaluation by Experts

The realistic simulator was tested by 12 veterinarians. The group consisted of nine members of staff from the Clinic for Cattle of the TiHo and three external practicing veterinarians in the field of cattle medicine. The experts performed the operation independently on the realistic simulator. There was no training or OSCE for verification. Thus, they were not part of the blended-learning/face-to-face study.

### 2.4. Data Acquisition

#### 2.4.1. Questionnaire

The questionnaires for data collection were developed based on variants already validated for study purposes. Before use, all questionnaires were checked by the CSL and E-Learning Consulting of the TiHo, with a focus on form, didactics, comprehension, and time taken to complete.

Three different questionnaires were used in the study of clinical-year students (see Figure 5), and another questionnaire was used for the simulator evaluation by the experts. 

In addition to free text responses, closed questions with rating scales were used. Likert-type questions with a four-point scale (1 = “Strongly disagree”, 2 = “Somewhat disagree”, 3 = “Somewhat agree”, 4 = “Strongly agree”, and 0 = “I don’t know”) were used as the main question type. Furthermore, the following question types were applied: school grades (1 = very good, 2 = good, 3 = satisfactory, 4 = sufficient, 5 = poor, and 6 = insufficient), percentages between 0 and 100 percent, checkbox questions with single-choice, and questions with multiple-choice.

Each questionnaire included a privacy statement, as well as the specification of an individual code, which made it possible to assign pairs of questionnaires at different points in time. The questions that followed were grouped into subsections according to the study group and time:

The demographic data registered the profession, field of expertise, professional experience of the experts, and the evaluation of one’s own abilities in relation to the topic of AD.

The subsection practical experience regarding the surgical treatment of AD in cattle was asked in both experimental groups (CY, experts). The role (surgeon or assistant), number, and frequency of operations were recorded, as well as the method(s) used.

The self-assessment of skills was determined at different points in time in the CY study (see Figure 5). It was asked before the study (t1), after the group-dependent training (t2), and after the OSCE (t3). The probability of recognizing the structures rumen, abomasum displaced to the left, pylorus, and greater omentum during exploration was asked. Theoretical and practical knowledge were assessed using German school grades in descending order, from grade 1, the best school grade (“very good”) to the worst, grade 6 (“insufficient”). The core of self-assessment was the assessment of the surgical steps, as well as their execution with Likert-type response options. For example, “I am familiar with the procedure and sequence of steps for abomasal surgery for left-sided AD laparotomy using Dirksen’s method in cattle” or “I have confidence in my ability to puncture and degas the left-shifted abomasum correctly after localizing it”.

As part of the evaluation, the video training, Simulator 1 and Simulator 2, and their application were rated. The video training was evaluated in terms of quality, its embedding in the online platform, and its suitability as a training module. Simulators 1 and 2 were evaluated in terms of their appearance, haptics, anatomy, and integrated structures. While the experts judged whether the realistic simulator was suitable for performing the surgical method, the students rated the efficiency of the two simulators as training objects. All participants had the opportunity to make suggestions for improvement in the form of free text answers.

In the subsection on the use of simulators, the instructional tool (for example, Simulator 1, Simulator 2, or live animal) and forms of integration (for example, obligatory program in the CY or optional CSL courses) could be prioritized. Moreover, questions regarding the general criteria for simulations and their importance were asked (for example, realism or animal welfare).

Finally, the studies were evaluated by the students in terms of subject matter, implementation, supervision, and time frame.

#### 2.4.2. Objective Structured Clinical Examination (OSCE)

The learned practical skills in the CY study were examined in the form of an OSCE station format compiled by the TiHo [26].

Objective verification was guaranteed by a checklist (see Supplementary Document S5). This was validated in form and content before use [26]. The 31 items structured the surgical technique chronologically in small steps. In addition to the steps of abomasal surgery, the order of the substeps, the handling of the instruments, and an antiseptic working practice were highlighted.

The scoring was performed in the categories “fulfilled” and “not fulfilled”. Items that were shown completely and correctly were considered “fulfilled”. For some items, the category “partially fulfilled” could be scored [26]. Skills that could not be demonstrated within the 25-min time limit were scored as “not fulfilled”. The evaluation was performed by independently trained teaching staff.

Each checklist was marked with an individual code to assign it to the questionnaire pairs.

### 2.5. Statistical Analyses

A data analysis was performed using SAS software, version 9.4; and SAS^®^ Enterprise Guide^®^ 7.15 (SAS Institute Inc., Cary, NC, USA). A descriptive analysis and graphic creation were also run with Microsoft^®^ Office Excel 2016 (Microsoft Corporation, Redmond, WA, USA). The *p*-values below 0.05 were assumed to be significant.

#### 2.5.1. Study with Students in Their Clinical Year

The OSCE results of the CY students were analyzed with descriptive statistics. The influence of the experience and group (video training and face-to-face training) on the OSCE results was evaluated, and both were compared using a linear model. This incorporated unequal variances depending on the experience of the student. For the post hoc comparisons of means, the Tukey–Kramer adjustment was used.

The first self-assessment of skills questions was displayed graphically by descriptive statistics. The following “surgical substeps” questions (see Supplementary Document S1–S3) of self-assessment of skills were added to the self-assessment score, using the Likert scale: “Strongly disagree” equals value 1, “Somewhat disagree” equals value 2, “Somewhat agree” equals value 3, and “Strongly agree” equals value 4. Statements with “I don’t know” were considered with value 0. Based on the responses, the median, mean, minimum, maximum values, and standard deviation were calculated before the study (t1), after the video and face-to-face training (t2), and after the OSCE (t3). The influence of experience and group on the self-assessment scores at t2 were analyzed using the same linear model methods as the OSCE results.

A Wilcoxon signed-rank test was used to determine whether the self-assessment and the probability of identifying abdominal structures were significantly different before (t1) and after the study (t3).

In order to assess whether the study participants could realistically assess themselves, the self-assessment subscore was compared with the OSCE score after the training or before the OSCE. Therefore, the correlation and linear regression between self-assessment of skills at this time (t2) and the OSCE score were analyzed.

A dichotomization was used to test individual subscales of self-assessment of skills: questions about “surgical substeps” (except the first three questions) in Questionnaire II CY (see Supplementary Document S2) were coded bimodally. “Strongly disagree” and “Somewhat disagree” equal 0, and “Somewhat agree” and “Strongly agree” equal 1. No participant selected the option “I don’t know”. The corresponding items were then assigned to the OSCE checklist, and subtotals were calculated. Subtotals were also coded bimodally: within item groups, scores greater than or equal to 60% were coded as 1, and scores less than 60% were coded as 0. The threshold was 60%, as this corresponded to the TiHo scoring scheme. Accordingly, a (partial) skill is considered successfully completed/passed at 60% (absolute pass mark). It was assessed whether participants who were self-confident in performing part of the operation were also able to demonstrate the skill correctly in the OSCE.

#### 2.5.2. Evaluation Analysis

Evaluation results were analyzed descriptively and shown graphically. Likert-type questions were represented with diverging bar charts.

## 3. Results

### 3.1. Study with Students in Their Clinical Year

In the study with students in their clinical year, 21 pairs of questionnaires including OSCE could be interpreted. The distribution of the students is displayed in Table 1.

#### 3.1.1. Evaluation of Practical Training versus Video Training

At the end of the study, students evaluated the traditional face-to-face teaching method (Group 1) and the alternative blended learning format (Group 2).

**Figure 6:**
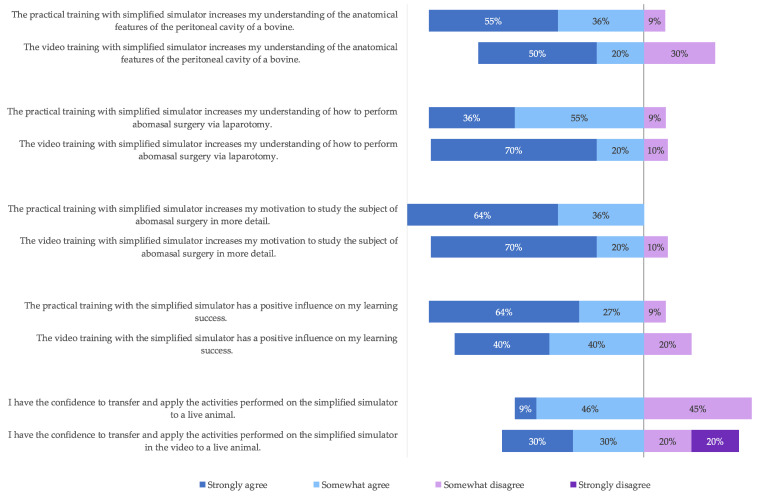
Comparison between video training and practical training.

The survey (Figure 6) showed that the majority of students improved their (self-rated) anatomical orientation in the peritoneal cavity, their (self-rated) understanding of abomasal laparotomy procedures, and their (self-rated) learning success in this regard, regardless of the group. The group that completed the practical training was slightly more able to agree with the statements listed in Figure 6. In both groups, just over half of the students were (self-rated) confident in applying the skills learned in the classroom or video training to a live animal. However, in the video group, 20% of the group participants did not agree at all with the application to a live animal.

The majority of students (90%) rated the quality of the video training (e.g., presentation of the simulators or visibility of the skills shown) as good and the Moodle learning platform as well suited for implementing video training. All participants in the video group stated that they understood what they were supposed to learn from the video. In the form of free text answers, (more) images and videos of abomasal surgery on live animals were requested (n = 2). It was also suggested to integrate the results of the medical anamnesis (n = 1) and to test the knowledge of organ identification in the form of questions (n = 1).

#### 3.1.2. Objective Structured Clinical Examination (OSCE)

A maximum of 76 points could be achieved in the OSCE. The lowest score was 50% (38 points), and the highest score was 94% (71.5 points). Only 5 of the 21 students failed the OSCE, achieving less than 60%: 2 students from the face-to-face group and 3 from the video group. The results depending on the group and experience are presented in Table 2.

The OSCE results were used to evaluate the effectiveness of group-based training. Table 3 shows the effects of group and experience in relation to the attestation.

It can be concluded that neither group, experience alone, nor their interactions led to different OSCE results. Despite the mean difference of 7.15 percentage points in OSCE, the performance of students who completed the video training was not significantly different from those who completed face-to-face practical training.

#### 3.1.3. Self-Assessment of Skills 

The self-assessment of skills was determined at three time points: Before the study (t1), after the group-dependent training before the OSCE (t2), and after the OSCE (t3). The self-assessment score at time t2 is summarized in Table 4.

The effect of group and experience on the self-assessment score after the group-dependent training is shown in Table 5.

Similar to the study of group and experience on the OSCE result, the analysis of self-assessment at the post-training time point indicates that the group affiliation did not lead to a different self-assessment of skills. Accordingly, the self-assessment score t2 was about 30 points for both groups. In contrast to the OSCE, (in-)experience led to a different self-assessment of skills (*p*-value 0.0031). Experienced students assessed themselves at time t2 with 32 points, with a median of about 4 points better than inexperienced students.

**Figure 7:**
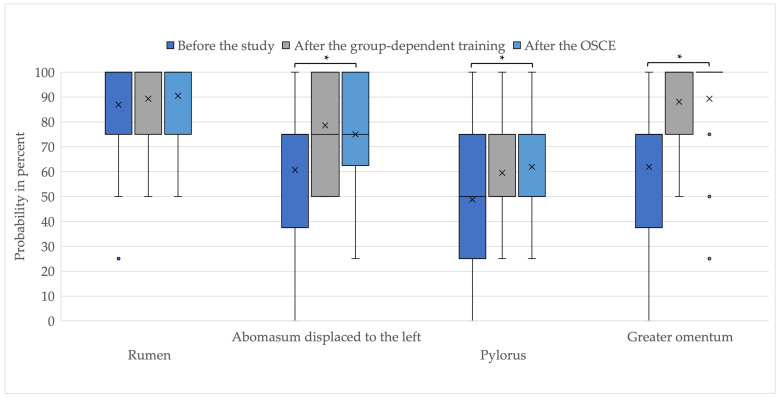
Self-assessment in identifying abdominal structures; * *p*-value below 0.05; x = mean value; ● = outliers.

The self-assessment in identifying the abdominal structures—rumen, left-sided displaced abomasum, pylorus, and greater omentum—increased on average in the study participation (Figure 7). The improvement in identifying left-sided displaced abomasum, pylorus, and greater omentum before the study and after the OSCE was significant.

Theoretical and practical knowledge were also improved by the group-dependent training and the OSCE. The median shows that students rated themselves as having a theoretical knowledge of German school-grade 3 and a practical knowledge of German school-grade 4 before the study. After the study, the median shows that students rated themselves one grade better.

Regardless of the effect size of the experience and group, the self-assessment score regarding performing surgery for left-sided AD using the Dirksen method increased on average from 23.1 points (t1) to 29.4 points (t2) to 30.2 points (t3). The increase in self-assessment of skills by a mean of seven points from t1 to t3 was significant with a *p*-value of less than 0.0001. The increase within the experienced/inexperienced group from t1 to t3 was also significant. The maximum score achieved was 36, which was also the maximum achievable score. Both the experienced and the inexperienced students increased their self-assessment of skills over the course of the study. The inexperienced study participants increased from a mean of 17.4 points before the study to 26.8 points after training and 27.2 points after the OSCE. The experienced participants started at a mean of 27.4 points pre-study. After the training, the average was 31.4 points, and after the OSCE, it was 32.4 points.

#### 3.1.4. Correlation of Self-Assessment of Skills and OSCE Result

The self-assessment score at time t2 after group-dependent training before OSCE was tested for its correlation with the OSCE result. Pearson’s correlation coefficient was 0.58121 and significant at 0.0057. The correlation analysis shows that high self-assessment scores coincided with high OSCE results. A linear regression was used to further quantify that an increase in the self-efficacy score by one point increased the OSCE results by around 1.5 points.

To control for the realistic self-assessment of skills, individual items of the OSCE checklist were further compared to individual self-assessment questions at time t2. The following statements of the students were checked:

- “I have confidence in my ability to select the appropriate needle and suture material for fixating the plate”.
- “I have confidence in my ability to differentiate between the abomasum and the rumen when exploring the peritoneal cavity”.
- “I have confidence in my ability to puncture and degas the left-shifted abomasum correctly after localizing it”.
- “I have confidence in my ability to reposition the abomasum correctly on my own”.
- “I have confidence in my ability to suture the plate correctly on my own”.
- “I have confidence in my ability to fix the plate correctly on my own using a button”.

All 21 students selected the correct suture material and needle for fixation of the abomasum in the OSCE. Of these, 20 students (95.24%) stated before the OSCE that they could select the needle and suture material. Only one inexperienced student was not confident in this skill, but accomplished it in the OSCE.

A total of 19 of 21 (90.48%) students were able to differentiate between abomasum and rumen during the exploration and were also able to correctly identify the two organs in the OSCE. Only two experienced students were unable to differentiate between the two organs correctly, although they were confident in doing so.

For the puncture and degassing of the abomasum, 18 of the 21 students (85.71%) rated themselves realistically: 17 students were able to demonstrate the skill correctly in the OSCE, and 1 subject did not correctly perform the puncture with degassing. Two inexperienced students stated that they were not confident in their ability to correctly ablate the abomasum but were able to perform the skill correctly in the OSCE. Only one experienced student was unable to perform it correctly, although having the confidence to do so.

For repositioning the abomasum, self-assessment of skills and OSCE matched in the case of 16 of the 21 students (76.19%): 12 reported being able to master the skill and also demonstrated this in the OSCE, while 4 estimated not to be able to reposition it correctly and also did not manage to do so in the OSCE. Three participants (one experienced and two inexperienced) repositioned it successfully, although they were not confident in the skill. Two participants (one experienced and one inexperienced) believed themselves to be able to master the skill but did not show correct repositioning of the abomasum in the OSCE.

A total of 16 of the 21 students (76.19%) had a realistic self-assessment of skills and correct OSCE performance in terms of plate fixation in the greater omentum. Three inexperienced students reported not being confident in this skill, but showed correct plate fixation during the OSCE. Two participants (one experienced and one inexperienced) did not show correct fixation of the plate, although they were previously confident in this substep of the op.

In the step “correct fixation of the button”, 18 of the 21 students (85.71%) assessed themselves realistically: 17 were confident in the skill and demonstrated this in the OSCE, and 1 student did not believe he/she was able to master the skill and did not show correct fixation of the button in the OSCE. Three students (one experienced and two inexperienced) managed to perform the skill correctly in the OSCE, although they did not believe they could do it beforehand.

### 3.2. Evaluation of the Simulators

#### 3.2.1. Simplified Simulator

The simplified simulator was tested by CY students as a practical (Group 1) and video-based (Group 2) training object in preparation for abomasal surgery. The evaluation results are shown in Supplementary Figure S1. The appearance of the simplified simulator was rated by the 21 students dichotomously as (somewhat) realistic and (somewhat) unrealistic. Regarding the appearance, the respondents stated that the box should be opaque (n = 3), and the organs should have a more realistic color (n = 1). Also noted was the improved representation of the tuber coxae (n = 1). The majority of the participants who used the simulator in face-to-face training stated that the simulator felt realistic. What they wished for was a more realistic surgical entrance (n = 2). Only 9% of the practical group students stated that important structures for performing abomasal surgery were missing in the simplified simulator. Suggestions for improvement included the addition of the following organs: liver (n = 5), reticulum (n = 4), kidney (n = 2), and uterus (n = 1). Regarding the item “Other”, it was suggested to fix the box more tightly (n = 2). Furthermore, suturing the plate and button directly to the simulator rather than to suture pads was requested (n = 1).

#### 3.2.2. Realistic Simulator

The realistic simulator was used by the students in the OSCE and evaluated afterwards (Supplementary Figure S2).

The study group of experts performed exploration and surgical techniques in left-sided AD by using the Dirksen method on the realistic simulator. Based on this, the simulator was evaluated as shown in Supplementary Figure S3.

All 21 students and 11 of the 12 experts strongly agreed or somewhat agreed that the simulator looked realistic. A more realistic coloring of the organs was desired, especially of the abomasum with pylorus and/or intestine (n = 6). A total of 81% of the students and 11 of the 12 experts somewhat agreed or strongly agreed that the haptics was realistic. It was noted that the use of other materials could provide better haptics and organ identification (n = 6). In particular, improvements in weight, texture, and/or wall thickness were desired for the abomasum, greater omentum, reticulum, and intestine (n = 10). A multi-layered representation of the abdominal wall was further noted as an improvement (n = 1). At 95%, the majority of students confirmed that the realistic simulator improved their understanding of the anatomical features of the bovine peritoneal cavity. All students and experts indicated that all important abdominal structures for performing abomasal surgery were included, whereas incorporating a bony pelvis was listed as a suggested improvement by experts (n = 4). In addition, integrating a separate skin layer in the region of the button wound was suggested (n = 2). During exploration, it was noted that the reticulum could be positioned slightly more ventrally (n = 4).

The student opinion survey shows that the realistic simulator increased the understanding and learning success of the majority of the students. In this context, 81% were fully confident or were somewhat confident in transferring and applying the skills they had learned to a live animal.

All experts considered the realistic simulator to be completely or somewhat suitable for performing abomasal surgery by laparotomy.

#### 3.2.3. Application of the Simulators

Of the 21 students, 70% desired integration of the developed simulators in the form of an elective course, and 60% desired integration in the form of a CSL course. All CY students supported the integration of the exercise in their clinical year.

The desired practice objects for learning and practicing an abomasal surgery are shown in Table 6. The realistic simulator received the most support from both the 21 students (80%) and the 12 experts (67%). Live animals were requested as a training object by 75% of the students. Slightly less than half (40%) of the students desired a combined use of the simplified simulator, realistic simulator, and video training. No study participant favored discontinuing simulation-based training in the future.

## 4. Discussion

In this educational study, a simplified simulator and a realistic simulator were developed at the CSL for learning the surgical technique for left-sided abomasal displacement, using the Dirksen method. They were evaluated for suitability and future use. No comparable simulator has yet been described in the current literature. To test the simulators, the blended learning method was compared to traditional face-to-face teaching in a pilot study. Students’ self-assessment of skills was also assessed.

Blended learning is characterized by a combination of online and face-to-face learning [8,10,27]. This combination could be called an emerging teaching method and was already tested several times in medicine [12,13,14,15]. At the Clinic for Ruminants at the University of Veterinary Medicine Vienna (VUW), Austria, an additional course was created in which students could work on the topic of endoscopy in ruminants [12]. Videos, pictures, and quizzes were used to illustrate the topic. According to Bernkopf et al. [12], this improves the quality of teaching. Supplementary video-based training sessions can increase knowledge growth [14,15,28]. At the University of Pennsylvania, USA, a study by Levitan et al. [14] used a 26-min laryngoscopy instructional video for learning intubation. The face-to-face group, which received no online preparation, achieved a mean initial success rate of 46.7%. On the other hand, the video group in the blended learning format had an average initial success rate of 88.1%. Consequently, the learning outcome was increased by 41.4 percentage points [14]. The transferability of these successes to complex surgical skills should be tested in the present pilot study and compared to traditional face-to-face teaching.

The results show that there was no significant difference (*p*-value 0.2102) in OSCE performance between the video group and the face-to-face group. Regarding the different methods of preparation for abdominal surgery, the students’ opinions in the present study show that both video and face-to-face training, as well as in-person application, were predominantly positively rated. The results provide an indication that the blended learning method can be suitable for the simulator-assisted learning of complex clinical skills and is well accepted by students. It should be noted that the video could be viewed several times. However, the practical face-to-face training took place only once. 

Martinsen and Jukes [29] suggest that simulations promote the development of solution strategies and clinical thinking, especially when multimedia systems are used in blended learning and when subject content is also linked. The students’ opinion in this paper shows that material for asynchronous preparation should be as interactive as possible. With the help of the video in the present study, all students were able to understand what needed to be practiced. Students welcomed the interactivity of integrated imagery, additional information, and questions. It was shown that information material should be processed in a broad context, for example, by integrating preliminary reports and diagnostic results into clinical scenarios. The modules integrated into Moodle, using H5P, were rated as suitable by 90% of the students and are therefore recommended.

Furthermore, Martinsen and Jukes [29] describe that online simulations are an appropriate tool but should be complemented by practical experience. This can be confirmed, as according to that study, online-only instruction is not recommended for complex veterinary skills. The student survey showed that only half of the students were confident in applying the skills learned in the video training to a live animal. It should be noted that the transferability of the skills to live animals was not tested in the present study.

Several studies have demonstrated that simulator-based training increases learning and is superior to face-to-face instruction only [18,19,30,31,32]. Baillie et al. [31] and Giese et al. [32] used the example of the bovine rectal palpation to show that not only high-fidelity but also mid-fidelity simulators increase first-day skills. In a study by Brombacher-Steiert et al. [19], the mid-fidelity simulator for transrectal ultrasonography in cattle was able to determine significantly higher self-efficacy after simulator-based training [19]. Similar increases in self-efficacy through simulator-based training are described in various studies [18,20,21,22,23,24,25,30].

The term self-efficacy was defined by Bandura [17] and describes the extent to which people believe they are competent to perform certain activities. This assessment has a significant impact on feelings, thoughts, and actions [16]. Evidence shows that high self-confidence in relation to specific skills, but also in general, is associated with a high success rate [16,17]. In the present pilot study, despite the high complexity of the surgery, an increase in self-assessment of skills was achieved. On average, the probability of identifying the greater omentum, the left displaced abomasum, and the pylorus increased significantly by 27.38 percentage points, 14.29 percentage points, and 13.1 percentage points, respectively. In addition, students rated their practical knowledge one German school-grade higher after the study. The self-assessment of skills increased significantly. Since the group did not lead to different self-assessment and OSCE scores, the data provide an indication that blended learning can be a suitable alternative to face-to-face simulator training in terms of increasing students’ self-assessment of skills.

While self-overestimation in daily life can result in personal development and success, accurate self-assessment of skills is essential in healthcare professions. The reason for this is the fatal consequences for patients in the case of misjudgments [16,17,33]. In the study by Schneider [18], it was demonstrated that self-efficacy is higher than objective performance, and especially inexperienced students overestimate themselves. The general overconfidence could be refuted in our study. The results indicate that high self-assessment scores correlate with high OSCE scores. To further differentiate this result, individual surgical steps were compared with the corresponding self-assessment items. This shows that the correct self-assessment of skills varies within surgery steps; for example, while the students were able to assess themselves correctly in selecting the suture material (95.24%) and identifying the rumen and abomasum (90.48%), the percentage was lower for repositioning the abomasum and suturing the plate (76.19% each). This may be due to the increased difficulty and complexity of the latter two skills.

The simulators developed were evaluated and assessed for their future use in teaching. Simulators can be categorized as low-fidelity, mid-fidelity, and high-fidelity simulators [34,35]. The simplified simulator developed in the present study was made from everyday materials and thus belongs to the category of low-fidelity simulators, while the life-size realistic simulator belongs to the category of mid-fidelity simulators.

According to Samia et al. [36], the suitability of simulators can be assessed with “face validity”, “content validity”, “construct validity”, “concurrent validity”, and “predictive validity”. Due to the simple materials, abstract colors, and constant visibility of the simulated organs, the simplified simulator was only able to convince half of the students in the category “appearance” (face validity). However, it had the advantage of allowing students to visualize the anatomy of the bovine peritoneal cavity. Despite simple materials such as fabric and elastic, the simulator was able to convince 82% of the students with a (somewhat) realistic feeling. The majority of students (100% of the video group and 91% of the face-to-face group) also reported that all important anatomical structures for performing abomasal surgery were included (content validity). This result shows that the key points of a simulation can be performed with simplified simulators that are limited to the essentials. Simulator 1 was intentionally kept simplified, schematic, and transparent to provide a visual representation of abdominal anatomy. Nevertheless, it is not surprising that the more realistic simulator, with its more comprehensive features, received better ratings: it was visually convincing (face validity) to all students and 82% of the experts. Only haptics was rated similarly to Simulator 1. This is due to similar abstract materials. This simplification, as also described in the study by Brombacher-Steiert et al. [19], allows for the technique to be learned on a live animal without confounding factors (e.g., defensive movements of the animal, fatty greater omentum, and size of the animal). In terms of the content validity of the peritoneal cavity, Simulator 2 impressed all students and experts. It also included all of the organs that students requested in their free-text responses for Simulator 1. The additional organ replicas not only allowed the surgical technique to be performed but also allowed an almost complete exploration of the peritoneal cavity. According to the students, the simulator thus increased their motivation, understanding, and learning success. Therefore, it was well accepted by the students as a training object. The suitability of Simulator 2 for learning and performing abdominal surgery was affirmed by all experts. Thus, Simulator 2 can be considered a suitable alternative to live animals for learning and consolidating the exploration and surgical technique of Dirksen in left-sided AD.

In summary, the suitability of the simulators can be verified. In the future, the simulators can be used in combination according to the students’ preferences. The preparation for the course can be performed asynchronously with Moodle by integrating the video created for the present study. This study indicates that blended learning can be a suitable alternative to face-to-face teaching only.

In addition, the combination of the realistic simulator with the opening and closing of the peritoneal cavity is a good way to practice the complete abomasal surgery. Studies show that combined learning stations can add significant value [18]. In human medicine, complex medical skills such as operations are often performed as a team, with clinicians or students from different semesters [37,38,39]. Teamwork and communication are part of the training, as well as the skills themselves [39]. Such a surgery simulation would also be conceivable and useful with the realistic simulator.

### Limitations and Strengths

It has to be noted that, with a total of 21 students, the sample size in this pilot study was relatively small. One challenge was that only clinical-year students from one clinic were available to participate, and the study had to fit into the existing schedule. Expecting different results when planning sample sizes was another problem. Further evaluation with more students is recommended, especially since not all students did the course at the same time. The students were batched according to their clinical year. Due to the small sample size, a possible “batch effect” was not incorporated into the analysis. For this reason, the results of this study have less statistical strength due to the small number of test subjects. Additional studies are needed to further evaluate blended learning in order to confirm the results of this pilot study and to be able to fully describe the effect of blended learning. The inclusion of students from other clinics and/or years of study would be recommended, as only students from the Clinic for Cattle in their clinical year took part in this pilot study. Nevertheless, this pilot study showed a trend that blended learning can be a suitable alternative to traditional face-to-face teaching clinical skills. Other studies, which cite their relatively small number of participants as a limitation, also describe that results cannot be generalized, but that trends can be identified [18,19,40]. We hope that this work will provide the groundwork for further studies in veterinary education research.

## 5. Conclusions

Simulator-based training enables students to learn complex surgical skills, such as the surgery technique for left-sided abomasal displacement, using the Dirksen method in a protected learning environment. The results indicate an increase in students’ self-assessment of skills, knowledge, learning, and motivation. The present pilot study indicates that blended learning can be a suitable alternative to traditional face-to-face teaching clinical skills. Further research is needed to support the use of blended learning in the veterinary education of skills.

## Figures and Tables

**Table 1:** Distribution of participants among groups and their level of experience.

	Inexperienced	Experienced	Total
Group 1 *	5	6	11
Group 2 *	4	6	10
Total	9	12	21

* Group 1, practical training; Group 2, video training.

**Table 2:** OSCE results depending on the group and experience.

Group *	Experience **	OSCE Result Mean Value	OSCE Result Standard Deviation	OSCE Result Median	OSCE Result Minimum	OSCE Result Maximum
1		76.43%	15.11	80.26%	50%	94.08%
2		69.28%	10.45	68.10%	55.26%	84.87%
	0	67.54%	13.08	70.39%	50%	84.87%
	1	77.14%	12.40	78.95%	55.26%	94.08%
1	0	66.18%	14.07	70.39%	50%	84.21%
1	1	84.98%	10.27	86.84%	68.42%	94.08%
2	0	69.25%	13.61	67.77%	56.58%	84.87%
2	1	69.30%	9.25	68.10%	55.26%	80.92%

* Group 1, practical training; Group 2, video training; ** Experience 0 = inexperienced; Experience 1 = experienced.

**Table 3:** Effects of group and experience on OSCE results.

Effect	*p*-Value
Group	0.2102
Experience	0.0685
Group × Experience	0.0700

**Table 4:** Self-assessment score after the group-dependent training (t2) depending on group and experience.

Group *	Experience **	Score t2 Mean Value (Points)	Score t2 Standard Deviation	Score t2 Median (Points)	Score t2 Minimum (Points)	Score t2 Maximum (Points)
1		29	3.97	30	22	34
2		29.9	3.73	30	22	35
	0	26.8	3.6	28	22	31
	1	31.4	2.57	32	27	35
1	0	26.4	3.85	27	22	31
1	1	31.2	2.71	31	27	34
2	0	27.3	3.77	28	22	31
2	1	31.7	2.66	32.5	28	35

* Group 1, practical training; Group 2, video training; ** Experience 0 = inexperienced; Experience 1 = experienced.

**Table 5:** Effect of experience and group on self-assessment score after the group-dependent training (t2).

Effect	Group *	Experience **		Group *	Experience **	*p*-Value ***
Group *	1		Compared with	2		0.6190
Experience **		0			1	**0.0031**
Group × Experience	1	0		1	1	0.0743
Group × Experience	1	0		2	0	0.9812
Group × Experience	1	0		2	1	**0.0435**
Group × Experience	1	1		2	0	0.2265
Group × Experience	1	1		2	1	0.9844
Group × Experience	2	0		2	1	0.1483

* Group 1, practical training; Group 2, video training; ** Experience 0 = inexperienced; Experience 1 = experienced; *** *p*-values are adjusted using the Tukey–Kramer method; *p*-values below 0.05 are considered significant (bold).

**Table 6:** Desired application of the simulators.

Desired Training Object	Students	Experts
Simplified simulator	15%	-
Realistic simulator	80%	67%
Combination of simulators with video	40%	-
No application of the simulators	0%	0%
Live animal	75%	42%

## Data Availability

The data presented in this study are available on request from the corresponding author. The data are not publicly available due to data privacy.

## Supplementary Materials

The following supporting information can be downloaded at https://www.mdpi.com/article/10.3390/ani13243822/s1, Document S1: Translation of the questionnaire I for students in their clinical year (CY); Document S2: Translation of the questionnaire II for students in their clinical year; Document S3: Translation of the questionnaire III for students in their clinical year; Document S4: Translation of the questionnaire for experts; Document S5: Translation of the Objective Structured Clinical Examination (OSCE) checklist; Figure S1: Evaluation of simplified simulator by students (Group 1: practical training; Group 2: video training); Figure S2: Evaluation of realistic simulator by students; Figure S3: Evaluation of realistic simulator by experts.
