# Supplementary material for: Simulator-Assisted Training of Abomasal Surgery—A Pilot Study Using Blended Learning and Face-to-Face Teaching
Source: Animals (Basel). 2023 Dec 11;13(24):3822. doi: 10.3390/ani13243822 (PMC10740769; doi:10.3390/ani13243822)
Supplement: Supplementary file 1 [file animals-13-03822-s001.zip › animals-2684157-supplementary.pdf]

Document S1. Translation of the questionnaire I for students in their clinical year (CY)

Questionnaire I Clinical year (CY) students - level of experience and self-efficacy

The following abbreviations are used below:
Abomasal displacement – AD
Operation – Op

Please tick the answer that applies the most to you. Unless otherwise indicated, the answer options are single choices. If you have marked an answer incorrectly, please fill out the box completely and place a new cross in the corresponding correct answer box.

01 | Practical experience regarding surgical treatment of abomasal displacement in cattle

		**Quantity**	**Applied methods**<br>(multiple answer options are possible)
	I have, as part of my veterinary studies up to now …		
**1.**	… assisted in surgical treatment of an AD by laparotomy (surgical opening of the peritoneal cavity) on a live animal in the following amount of times:	☐ 0 operations<br>☐ 1-5 operations<br>☐ 6-10 operations<br>☐ 11-20 operations<br>☐ >20 operations	☐ Dirksen *(right flank, abomasal attachment by means of plate and button)*<br>☐ Modified Dirksen method *(right flank, abomasal attachment only by means of plate)*<br>☐ Utrecht method *(laparotomy in the left flank)*
**2.**	… independently performed surgical treatment of an AD by laparotomy on a live animal under supervision in the following amount of times:	☐ 0 operations<br>☐ 1-5 operations<br>☐ 6-10 operations<br>☐ 11-20 operations<br>☐ >20 operations	☐ Dirksen *(see above)*<br>☐ Dirksen modified *(see above)*<br>☐ Utrecht method *(see above)*
**3.**	… assisted in endoscopic surgery of an AD on a live animal the following amount of times:	☐ 0 operations<br>☐ 1-5 operations<br>☐ 6-10 operations<br>☐ 11-20 operations<br>☐ >20 operations	☐ Janowitz (*puncture of the abomasum under endoscopic visual control with tilting of the cow, percutaneous fixation by toggle)*<br>☐ Christiansen *(puncture of the abomasum under endoscopic visual control, percutaneous toggle-pin fixation, suture deployment with spieker, cow remains standing)*
**4.**	… independently performed endoscopic surgery of an AD on a live animal under supervision the following amount of times:	☐ 0 operations<br>☐ 1-5 operations<br>☐ 6-10 operations<br>☐ 11-20 operations<br>☐ >20 operations	☐ Janowitz *(see above)*<br>☐ Christiansen *(see above)*

02 | Self-efficacy

**1.**	Rank the following treatments. Number 1 for the procedure you feel most confident about performing on your own and then in descending order to number 6 for the procedure you feel least confident about performing on your own.	… Castration of male cattle<br>… Tail amputation of cattle<br>… Cesarean section of cattle<br>… Op - left-sided AD by laparotomy in cattle<br>… Op - left-sided AD endoscopy in cattle<br>… Op - right-sided AD by laparotomy in cattle

		**0%**	**25%**	**50%**	**75%**	**100%**
**2.**	I can identify the rumen during laparotomy with the following probability.					
**3.**	I can identify the left-sided displaced abomasum during laparotomy with the following probability.					
**4.**	I can identify the pylorus during laparotomy with the following probability.					
**5.**	I can identify the greater omentum during laparotomy with the following probability.					

		**Grade 6**	**Grade 5**	**Grade 4**	**Grade 3**	**Grade 2**	**Grade 1**	**I don't know**
**6.**	I would rate my theoretical knowledge about AD and its treatment with the following German school grade.							
**7.**	I would rate my practical knowledge about AD and its treatment with the following German school grade.							

	Surgical substeps	Strongly disagree	Somewhat disagree	Somewhat agree	Strongly agree	I don’ t know
**8.**	I am familiar with the procedure and sequence of steps for abomasal surgery for left-sided AD laparotomy using Dirksen’s method in cattle.					
**9.**	I have the confidence to perform abomasal surgery for left-sided AD laparotomy using Dirksen’s method in cattle independently.					
**10.**	I have the confidence to perform abomasal surgery for left-sided AD laparotomy using Dirksen’s method in cattle under supervision.					
**11.**	I have confidence in my ability to differentiate between the abomasum and the rumen when exploring the peritoneal cavity.					
**12.**	I have confidence in my ability to puncture and degas the left-shifted abomasum correctly after localizing it.					
**13.**	I have confidence in my ability to reposition the abomasum correctly on my own.					
**14.**	I have confidence in my ability to suture the plate correctly on my own.					
**15.**	I have confidence in my ability to select the appropriate needle and suture material for fixating the plate.					
**16.**	I have confidence in my ability to fix the plate correctly on my own using a button.					

Thank you for participating!

Document S2. Translation of the questionnaire II for students in their clinical year

Questionnaire II CY students - Self-efficacy after group-dependent training

The following abbreviations are used below:
Abomasal displacement – AD
Operation – Op

Please tick the answer that applies the most to you. Unless otherwise indicated, the answer options are single choices. If you have marked an answer incorrectly, please fill out the box completely and place a new cross in the corresponding correct answer box.

01 | Self-efficacy

**1.**	Rank the following treatments. Number 1 for the procedure you feel most confident about performing on your own and then in descending order to number 6 for the procedure you feel least confident about performing on your own.	… Castration of male cattle<br>… Tail amputation of cattle<br>… Cesarean section of cattle<br>… Op - left-sided AD by laparotomy in cattle<br>… Op - left-sided AD endoscopy in cattle<br>… Op - right-sided AD by laparotomy in cattle

		**0%**	**25%**	**50%**	**75%**	**100%**
**2.**	I can identify the rumen during laparotomy with the following probability.					
**3.**	I can identify the left-sided displaced abomasum during laparotomy with the following probability.					
**4.**	I can identify the pylorus during laparotomy with the following probability.					
**5.**	I can identify the greater omentum during laparotomy with the following probability.					

		**Grade 6**	**Grade 5**	**Grade 4**	**Grade 3**	**Grade 2**	**Grade 1**	**I don't know**
**6.**	I would rate my theoretical knowledge about AD and its treatment with the following German school grade.							
**7.**	I would rate my practical knowledge about AD and its treatment with the following German school grade.							

	Surgical substeps	Strongly disagree	Somewhat disagree	Somewhat agree	Strongly agree	I don' t know
**8.**	I am familiar with the procedure and sequence of steps for abomasal surgery for left-sided AD laparotomy using Dirksen's method in cattle.					
**9.**	I have the confidence to perform abomasal surgery for left-sided AD laparotomy using Dirksen's method in cattle independently.					
**10.**	I have the confidence to perform abomasal surgery for left-sided AD laparotomy using Dirksen's method in cattle under supervision.					
**11.**	I have confidence in my ability to differentiate between the abomasum and the rumen when exploring the peritoneal cavity.					
**12.**	I have confidence in my ability to puncture and degas the left-shifted abomasum correctly after localizing it.					
**13.**	I have confidence in my ability to reposition the abomasum correctly on my own.					
**14.**	I have confidence in my ability to suture the plate correctly on my own.					
**15.**	I have confidence in my ability to select the appropriate needle and suture material for fixating the plate.					
**16.**	I have confidence in my ability to fix the plate correctly on my own using a button.					

Thank you for participating!

Document S3. Translation of the questionnaire III for students in their clinical year

Questionnaire III CY students - Evaluation and self-efficacy

The following abbreviations are used below:
Abomasal displacement – AD
Operation – Op

Please tick the answer that applies the most to you. Unless otherwise indicated, the answer options are single choices. If you have marked an answer incorrectly, please fill out the box completely and place a new cross in the corresponding correct answer box.

01 | Evaluation of the simplified simulator

		Strongly disagree	Somewhat disagree	Somewhat agree	Strongly agree	I don’t know
**1.**	I did the practical training on simulator 1.					
**2.**	The simplified simulator has a realistic look.					
**3.**	The simplified simulator has a realistic haptic.					
**4.**	The simplified simulator feels unrealistic.					
**5.**	The simplified simulator includes all important anatomical structures for performing abomasal surgery.					
**6.**	The simplified simulator increases my understanding of the anatomical features of the peritoneal cavity of a bovine.					
**7.**	The simplified simulator increases my understanding of how to perform abomasal surgery via laparotomy.					
**8.**	The simplified simulator increases my motivation to study the subject of abomasal surgery in more detail.					
**9.**	I have the confidence to transfer and apply the activities performed on the simplified simulator to a live animal.					
**10.**	Training with the simplified simulator has had a positive influence on my learning success with regard to performing an abomasal surgery independently.					
**11.**	In my opinion, the simplified simulator is better suited for teaching purposes than the realistic simulator.					
**12.**	In my opinion, the simplified simulator is less suited for teaching purposes than the realistic simulator.					

Need for optimization/suggestions for improving the simplified simulator:

- ☐ Further organs, for example:
- ☐ More realistic haptics, for example:
- ☐ More realistic optics, for example:
- ☐ Improved usability, for example:
- ☐ Other:

Further comments/ideas:

02 | Evaluation of the video training

		Strongly disagree	Somewhat disagree	Somewhat agree	Strongly agree	I don’t know
**13.**	I did the video training with simulator 1.					
**14.**	The video is of good quality.					
**15.**	The Moodle platform is well suited for video training.					
**16.**	By using the video training, I was easily able to understand what had to be practiced.					
**17.**	The simplified simulator shown in the video has a realistic look.					
**18.**	The simplified simulator shown in the video includes all important anatomical structures for performing abomasal surgery.					
**19.**	The simplified simulator shown in the video increases my understanding of the anatomical features of the peritoneal cavity of a bovine.					
**20.**	The simplified simulator shown in the video increases my understanding of how to perform abomasal surgery via laparotomy.					
**21.**	The simplified simulator shown in the video increases my motivation to study the subject of abomasal surgery in more detail.					
**22.**	In my opinion, the video training with the simplified simulator is sufficient preparation for performing an abomasal operation on a live animal.					
**23.**	The video training with the simplified simulator has had a positive influence on my learning success with regard to performing an abomasal surgery independently.					
**24.**	I have the confidence to transfer and apply the activities performed on the simplified simulator in the video to a live animal.					

Need for optimization/suggestions for improving the video training:

- ☐ Further organs, for example:
- ☐ More realistic optics, for example:
- ☐ More/other video interactions, for example:
- ☐ Improved usability, for example:
- ☐ Other:

Further comments/ideas:

03 I Evaluation of the realistic simulator

		Strongly disagree	Somewhat disagree	Somewhat agree	Strongly agree	I don’ t know
**25.**	The realistic simulator has a realistic look.					
**26.**	The realistic simulator has a realistic haptic.					
**27.**	The realistic simulator includes all important anatomical structures for performing abomasal surgery.					
**28.**	The realistic simulator increases my understanding of the anatomical features of the peritoneal cavity of a bovine.					
**29.**	The realistic simulator increases my understanding of how to perform abomasal surgery via laparotomy.					
**30.**	The realistic simulator increases my motivation to study the subject of abomasal surgery in more detail.					
**31.**	Training with the realistic simulator has had a positive influence on my learning success with regard to performing an abomasal surgery independently.					
**32.**	Training with the realistic simulator has had no influence on my learning success with regard to performing an abomasal surgery independently.					
**33.**	I have the confidence to transfer and apply the activities performed on the realistic simulator to a live animal.					

Need for optimization/suggestions for improving the realistic simulator:

- Further organs, for example:
- More realistic haptics, for example:
- More realistic optics, for example:
- Improved usability , for example:
- Other:

Further comments/ideas:

04 | Application of the simulators

		Strongly disagree	Somewhat disagree	Somewhat agree	Strongly agree	I don't know
**34.**	In my opinion, it is useful to practice surgery on a simulator before performing surgery on a live animal.					
**35.**	Performing abomasal surgery should be trained practically during the course of study as an obligatory course.					
**36.**	Performing abomasal surgery should be trained practically during the course of study as an elective course.					
**37.**	More practical training on the topic of abomasal surgery should be offered during the course of study.					
**38.**	I would like to see the realistic simulator combined with the opening and suturing of the peritoneal cavity.					
**39.**	I would like to see more simulators like the realistic simulator being an integral part of the veterinary medicine course of study in the future.					

		Multiple answer options are possible.
**40.**	Which instructional tool do you prefer for practicing abomasal surgery?	☐ Simplified simulator<br>☐ Realistic simulator<br>☐ Only video training<br>☐ A combination of video training and simplified simulator<br>☐ A combination of video training and realistic simulator<br>☐ A combination of video training, simplified and realistic simulator<br>☐ Living animal<br>☐ No practice<br>☐ Other: ______________________________
**41.**	In what way would you like to see simulators integrated into the course of study?	☐ As part of the clinical year<br>☐ As part of an elective course<br>☐ As part of a supervised Clinical Skills Lab (CSL) course<br>☐ Not being integrated in the course of study<br>☐ Other: ______________________________

	**In the case of a practical training tool what is important to me in general is:**	**Strongly disagree**	**Somewhat disagree**	**Somewhat agree**	**Strongly agree**	**I don’ t know**
**42.**	The realism (e.g. optics, haptics, inclusion of many relevant structures).					
**43.**	Animal welfare (e.g., practical training on live/dead animals).					
**44.**	Availability (e.g., storage, acquisition, delivery time).					
**45.**	Sustainability (e.g., plastic waste, reusability, resources consumed).					
**46.**	A stress-free environment.					
**47.**	Supervision (e.g. by a tutor).					
**48.**	Other: ____					

05 | **Evaluation of the study**

		Strongly disagree	Somewhat disagree	Somewhat agree	Strongly agree	I don’ t know
**49.**	I enjoyed participating in the study.					
**50.**	The organization was adequate.					
**51.**	Supervision by the tutor was adequate.					
**52.**	The time frame for the practical training/video training was appropriate.					
**53.**	The time frame for the Objective Structured Clinical Examination (OSCE) was appropriate.					

06 | **Self-efficacy**

**1.**	Rank the following treatments. Number 1 for the procedure you feel <u>most confident</u> about performing on your own and then in descending order to number 6 for the procedure you feel <u>least confident</u> about performing on your own.	… Castration of male cattle<br>… Tail amputation of cattle<br>… Cesarean section of cattle<br>… Op - left-sided AD by laparotomy in cattle<br>… Op - left-sided AD endoscopy in cattle<br>… Op - right-sided AD by laparotomy in cattle

		**0%**	**25%**	**50%**	**75%**	**100%**
**2.**	I can identify the rumen during laparotomy with the following probability.					
**3.**	I can identify the left-sided displaced abomasum during laparotomy with the following probability.					
**4.**	I can identify the pylorus during laparotomy with the following probability.					
**5.**	I can identify the greater omentum during laparotomy with the following probability.					

		Grade 6	Grade 5	Grade 4	Grade 3	Grade 2	Grade 1	I don't know
**6.**	I would rate my theoretical knowledge about AD and its treatment with the following German school grade.							
**7.**	I would rate my practical knowledge about AD and its treatment with the following German school grade.							

**Surgical substeps**		Strongly disagree	Somewhat disagree	Somewhat agree	Strongly agree	I don' t know
**8.**	I am familiar with the procedure and sequence of steps for abomasal surgery for left-sided AD laparotomy using Dirksen's method in cattle.					
**9.**	I have the confidence to perform abomasal surgery for left-sided AD laparotomy using Dirksen's method in cattle independently.					
**10.**	I have the confidence to perform abomasal surgery for left-sided AD laparotomy using Dirksen's method in cattle under supervision.					
**11.**	I have confidence in my ability to differentiate between the abomasum and the rumen when exploring the peritoneal cavity.					
**12.**	I have confidence in my ability to puncture and degas the left-shifted abomasum correctly after localizing it.					
**13.**	I have confidence in my ability to reposition the abomasum correctly on my own.					
**14.**	I have confidence in my ability to suture the plate correctly on my own.					
**15.**	I have confidence in my ability to select the appropriate needle and suture material for fixating the plate.					
**16.**	I have confidence in my ability to fix the plate correctly on my own using a button.					

Thank you for participating!

Document S4. Translation of the questionnaire for experts

Questionnaire experts

The following abbreviations are used below:
Abomasal displacement – AD
Operation – Op

Please tick the answer that applies the most to you. Unless otherwise indicated, the answer options are single choices. If you have marked an answer incorrectly, please fill out the box completely and place a new cross in the corresponding correct answer box.

01 | Demographics

		Multiple answer options are possible.
**1.**	Profession:	☐ Veterinarian<br>☐ Other: ______
**2.**	Field of expertise:	☐ Large animals (including cattle, cloven-hoofed animals and/or horses)<br>☐ Cattle<br>☐ Teaching<br>☐ Other: ______
**3.**	Professional experience:	______ years<br>☐ Veterinary specialist for ______<br>☐ Additional qualification in ______
**4.**	I would rate my practical knowledge of AD and its treatment as follows:	☐ Very good<br>☐ Good<br>☐ Satisfactory<br>☐ Sufficient<br>☐ Poor

02 | Practical experience regarding surgical treatment of abomasal displacement in cattle

<table>
<tr><th colspan="2"></th><th>Quantity</th><th>Applied methods
(multiple answer options are possible)</th></tr>
<tr><td colspan="4">As part of my job, I perform …</td></tr>
<tr><td>5.</td><td>… surgical treatment of an AD by laparotomy on a live animal independently in the following amount of times:</td><td>☐ daily
☐ weekly
☐ monthly
☐ yearly
☐ never</td><td>☐ Dirksen
(right flank, abomasal attachment by means of plate and button)
☐ Modified Dirksen method
(right flank, abomasal attachment only by means of plate)
☐ Utrecht method
(laparotomy in the left flank)
☐ Other: ____________</td></tr>
<tr><td>6.</td><td>… endoscopic surgery of an AD on a live animal independently the following amount of times:</td><td>☐ daily
☐ weekly
☐ monthly
☐ yearly
☐ never</td><td>☐ Janowitz
(p*uncture of the abomasum under endoscopic visual control with tilting of the cow, percutaneous fixation by toggle)*
☐ Christiansen *(puncture of the abomasum under endoscopic visual control, percutaneous toggle-pin fixation, suture deployment with spieker, cow remains standing)*
☐ Other: ____________</td></tr>
<tr><td colspan="4">I have, as part of my job up to now, ...</td></tr>
<tr><td>7.</td><td>... independently performed surgical treatment of AD by laparotomy on a live animal in the following amount of times:</td><td>☐ 0x
☐ 1-5x
☐ 6-20x
☐ 21-40x
☐ >40x</td><td>☐ Dirksen (*see above)*
☐ Modified Dirksen method (*see above)*
☐ Utrecht method (*see above)*
☐ Other: ____________</td></tr>
<tr><td>8.</td><td>... independently performed endoscopic surgery of an AD on a live animal the following amount of times:</td><td>☐ 0x
☐ 1-5x
☐ 6-20x
☐ 21-40x
☐ >40x</td><td>☐ Janowitz (*see above)*
☐ Christiansen (*see above)*
☐ Other: ____________</td></tr>
</table>

03 | Application of the simulators

		Multiple answer options are possible.
**9.**	Which simulator did you train on in today's course?	☐ Simplified simulator<br>☐ Realistic simulator<br>☐ On both simulators<br>☐ On neither simulator
**10.**	Which instructional tools do you prefer for practicing abomasal surgery?	☐ Simplified simulator<br>☐ Realistic simulator<br>☐ Only video training<br>☐ A combination of simplified and realistic simulator<br>☐ Living animal<br>☐ No practice<br>☐ I don't know
**11.**	In what way would you like to see simulators integrated into the course of study?	☐ As part of teaching in the veterinary medicine course of study<br>☐ As part of teaching in the context of further veterinary medicine training and postgraduate training<br>☐ As part of teaching in the veterinary medicine course of study <u>and</u> in the context of further veterinary medicine training and postgraduate training<br>☐ The simulators should not be used<br>☐ I don't know

		**Strongly disagree**	**Somewhat disagree**	**Somewhat agree**	**Strongly agree**	**I don’ t know**
**12.**	In my opinion, it is useful to practice surgery on a simulator before performing surgery on a live animal.					
**13.**	Performing abomasal surgery should be trained practically during the course of study as an <u>obligatory</u> course.					
**14.**	Performing abomasal surgery should be trained practically during the course of study as an <u>elective</u> course.					
**15.**	I would like to see more simulators like the realistic simulator being an integral part of the veterinary medicine course of study in the future.					

In the case of a practical training tool what is important to me in general is:		Strongly disagree	Somewhat disagree	Somewhat agree	Strongly agree	I don’ t know
**16.**	The realism (e.g. optics, haptics, inclusion of many relevant structures).					
**17.**	Animal welfare (e.g., practical training on live/dead animals).					
**18.**	Availability (e.g., storage, acquisition, delivery time).					
**19.**	Sustainability (e.g., plastic waste, reusability, resources consumed).					
**20.**	A stress-free environment.					
**21.**	Supervision (e.g. by a tutor).					
**22.**	Other: ______________________________					

04 | Evaluation of the simplified simulator

		Strongly disagree	Somewhat disagree	Somewhat agree	Strongly agree	I don’ t know
**23.**	I liked the training on the simplified simulator.					
**24.**	The simplified simulator has a realistic look.					
**25.**	The simplified simulator has a realistic haptic.					
**26.**	The simplified simulator feels unrealistic.					
**27.**	The simplified simulator includes all important anatomical structures for performing abomasal surgery.					
**28.**	The simplified simulator is suitable for performing abomasal surgery by laparotomy.					
**29.**	The simplified simulator increases my motivation to study the subject of abomasal surgery in more detail.					

Need for optimization/suggestions for improving the simplified simulator:

- ☐ Further organs, for example:
- ☐ More realistic haptics, for example:
- ☐ More realistic optics, for example:
- ☐ Improved usability, for example:
- ☐ Other:

Further comments/ideas:

05 | **Evaluation of the realistic simulator**

		Strongly disagree	Somewhat disagree	Somewhat agree	Strongly agree	I don' t know
**30.**	I liked the training on the realistic simulator.					
**31.**	The realistic simulator has a realistic look.					
**32.**	The realistic simulator looks unrealistic.					
**33.**	The realistic simulator has a realistic haptic.					
**34.**	The realistic simulator includes all important anatomical structures for performing abomasal surgery.					
**35.**	The realistic simulator is suitable for performing abomasal surgery by laparotomy.					
**36.**	The realistic simulator increases my motivation to study the subject of abomasal surgery in more detail.					

Need for optimization/suggestions for improving the realistic simulator:

- ☐ Further organs, for example:
- ☐ More realistic haptics, for example:
- ☐ More realistic optics, for example:
- ☐ Improved usability, for example:
- ☐ Other:

Further comments/ideas:

Thank you for participating!

Document S5 Translation of the Objective Structured Clinical Examination (OSCE) checklist

Checklist OSCE Surgery technique left-sided abomasal displacement

	**What the examinee should do:**	**Not fulfilled**	**Partially fulfilled**	**Fulfilled**	**Points**
**1.**	Select the correct suture material for the plate.				**2**
**2.**	Select the correct needle.				**1**
**3.**	Prepare threading of suture into the plate <u>before</u> threading into the needle.				**2**
**4.**	<u>Announcement:</u> Opening of the peritoneal cavity.				**1**
**5.**	Inserting the left hand into the peritoneal cavity and passing it caudally of the greater omentum towards the left flank.				**2**
**6.**	Correctly identifying the abomasum and rumen.				**3**
**7.**	Correctly inserting the degassing cannula up to the abomasum.				**4**
**8.**	Puncturing the gassed abomasum with the degassing cannula in an oblique direction.				**4**
**9.**	Announcement: Olfactory check at the degassing cannula.				
**10.**	Degassing the abomasum with left hand splayed out while permanently fixing the cone.				**3**
**11.**	Correctly removing the degassing cannula.				**4**
**12.**	Correctly cleaning the degassing cannula, no contact of cannula with sterile instruments on the operating table.				**2**
**13.**	Correctly repositioning the abomasum.				**4**
**14.**	Moving the greater omentum dorsally in the direction of the tuber coxae into the surgical field.				**2**
**15.**	Correctly identifying and naming the pylorus and the greater omentum.				**3**
**16.**	Identifying the correct plate position.				

	**Suturing the plate to the greater omentum.**				
**17.**	Stitching in a cranial to caudal direction with threaded plate through the greater omentum and then back in a caudal to cranial direction.				**3**
**18.**	Unthreading the suture and passing it through the outer holes and the central hole of the plate.				**3**
**19.**	Threading the suture and stitching through the greater omentum between caudal running sutures, then pulling tight.				**3**
	**Suturing the button.**				
**20.**	Correctly identifying position on the abdominal wall and indicating it from the outside.				**2**
**21.**	<u>Announcement:</u> Skin incision outside.				**1**
**22.**	Stitching the sutures from inside out, while protecting the needle with fingers in the peritoneal cavity.				**2**
**23.**	Passing sutures through the button two times.				**2**
**24.**	Making a visible check that no intestinal parts are/were knotted together.				**3**
**25.**	Tightening suture and tying with a surgical knot.				**2**
**26.**	Checking that plate fits tightly, resting close to the abdominal wall.				**3**
**27.**	Shortening suture.				**1**
**28.**	<u>Announcement:</u> Closing the wound with two interrupted horizontal mattress sutures and covering with a wound pad.				**1**
**29.**	<u>Announcement:</u> Closing the peritoneal cavity with a flank suture and covering with a wound pad.				**1**
**30.**	The clinical skills were performed in the correct order.				**3**
**31.**	Holding the scissors always in the ring finger-thumb grip.				**1**
**32.**	Aseptic procedures were followed for all skills.				**3**
	**Total**				

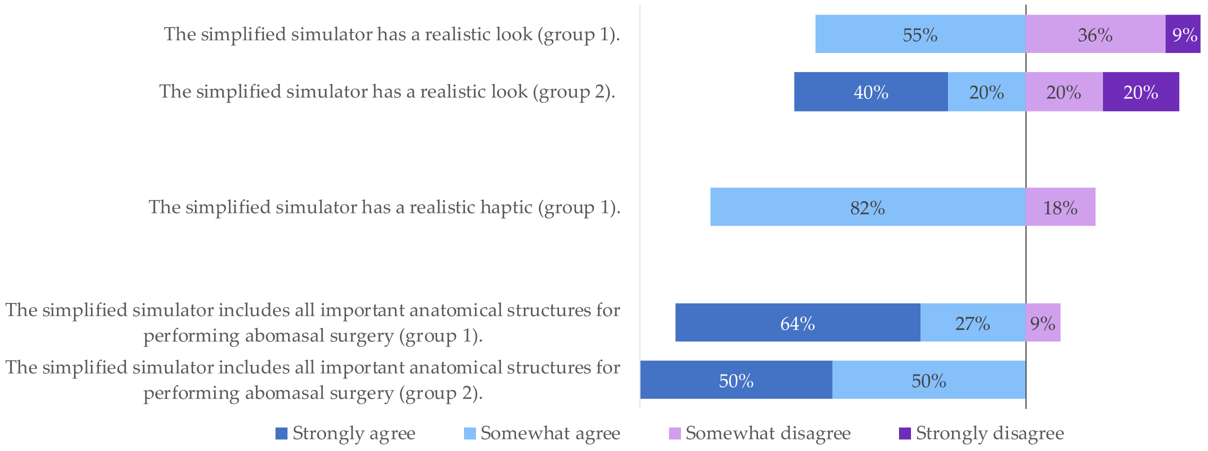

Figure S1. Evaluation of simplified simulator by students (Group 1: Practical training, Group 2: Video training).

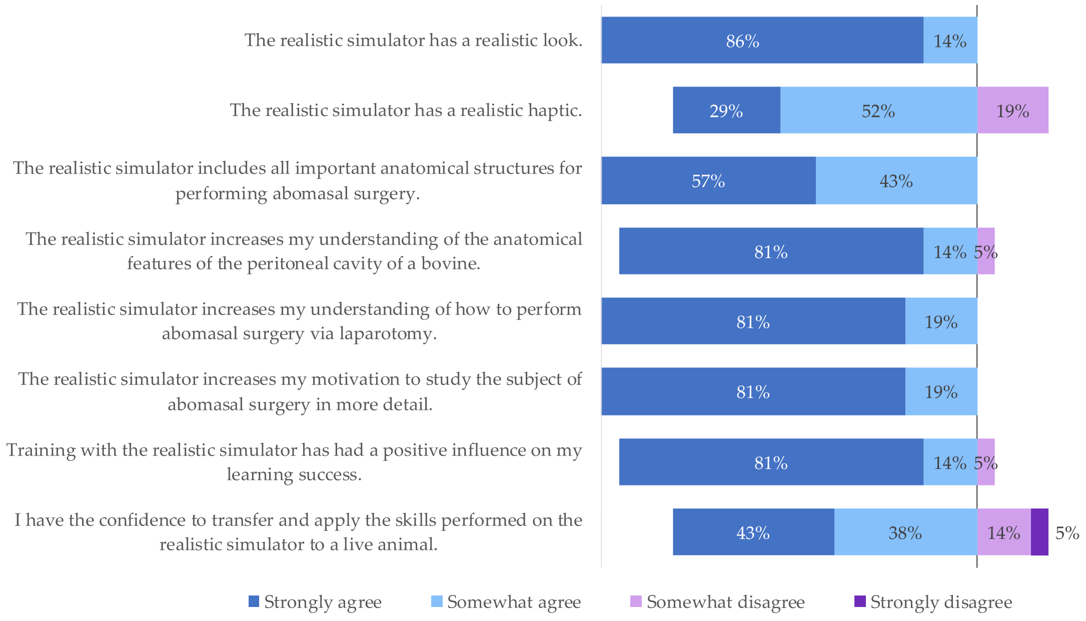

Figure S2. Evaluation of realistic simulator by students.

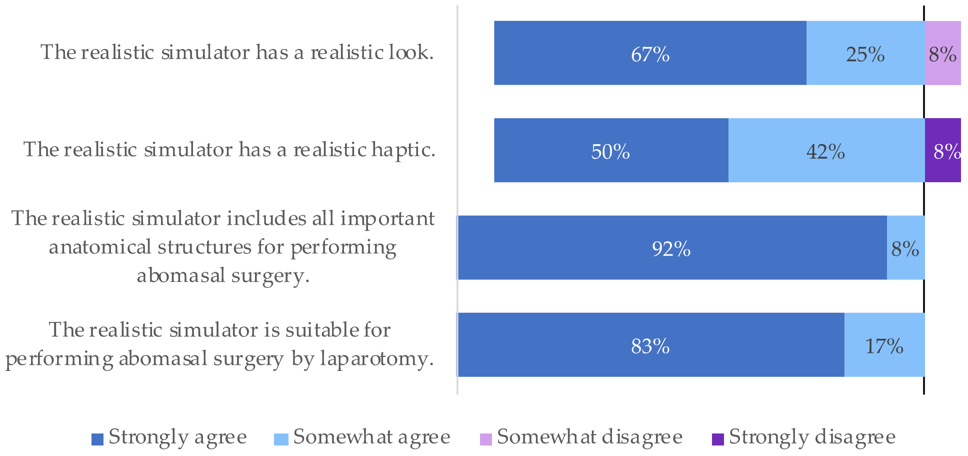

Figure S3. Evaluation of realistic simulator by experts.
